# Metabolomic analysis of rumen-protected branched-chain amino acids in primiparous dairy cows

**DOI:** 10.3389/fimmu.2024.1385896

**Published:** 2024-04-23

**Authors:** Zhikun Zhao, Jianan Dong, Dezhi Wang, Chen Zhao, Xu Tian, Yuan Meng, Yue Zou, Yang Zhao, Guixin Qin, Tao Wang, Zhe Sun, Xuefeng Zhang, Yuguo Zhen

**Affiliations:** ^1^ Key Laboratory of Animal Nutrition and Feed Science of Jilin Province, Jilin Agricultural University (JLAU)-Borui Dairy Science and Technology R&D Center, College of Animal Science and Technology, Jilin Agricultural University, Changchun, China; ^2^ Feed Engineering Technology Research Center of Ningxia Province, Ningxia Borui Science and Technology Co., Ltd., Yinchuan, China; ^3^ Postdoctoral Scientific Research Workstation, Feed Engineering Technology Research Center of Jilin Province, Changchun Borui Science & Technology Co., Ltd, Changchun, China; ^4^ College of Life Science, Jilin Agricultural University, Changchun, China

**Keywords:** branched-chain amino acids, oxidative stress, lactation performance, energy metabolism, metabolomics

## Abstract

**Introduction:**

Peripartal cows are susceptible to a negative energy balance due to inadequate nutrient intake and high energy requirements for lactation. Improving the energy metabolism of perinatal dairy cows is crucial in increasing production in dairy cows.

**Methods:**

In this study, we investigated the impact of rumen-protected branched-chain amino acid (RPBCAA) on the production performance, energy and lipid metabolism, oxidative stress, and immune function of primiparous dairy cows using metabolomics through a single-factor experiment. Twenty healthy primiparous Holstein cows were selected based on body condition scores and expected calving date, and were randomly divided into RPBCAA (n = 10) and control (n = 10) groups. The control group received a basal diet from calving until 21 d in milk, and the RPBCAA group received the basal diet and 44.6 g/d RPLeu, 25.14 g/d RPIle, and 25.43 g/d RPVal.

**Results:**

In comparison to the control group, the supplementation of RPBCAA had no significant effect on milk yield and milk composition of the dairy cows. Supplementation with RPBCAA significantly increased the concentrations of insulin, insulin growth factor 1, glucagon, and growth hormones, which are indicators of energy metabolism in postpartum cows. The very low density lipoprotein, fatty acid synthase, acetyl coenzyme A carboxylase, and hormone-sensitive lipase contents of the RPBCAA group were significantly greater than that of the control group; these metrics are related to lipid metabolism. In addition, RPBCAA supplementation significantly increased serum glutathione peroxidase and immunoglobulin G concentrations and decreased malondialdehyde concentrations. Liquid chromatography–mass spectrometry (LC-MS) analysis revealed 414 serum and 430 milk metabolic features. Supplementation with RPBCAA primarily increased concentrations of amino acid and lipid metabolism pathways and upregulated the abundance of serotonin, glutamine, and phosphatidylcholines.

**Discussion:**

In summary, adding RPBCAA to the daily ration can influence endocrine function and improve energy metabolism, regulate amino acid and lipid metabolism, mitigate oxidative stress and maintain immune function on primiparous cows in early lactation.

## 1 Introduction

Early lactation is crucial in the production cycle of cows. Dairy cows typically face considerable physiological challenges during the early stages of lactation. High lactation demand leads to endocrine disruption, and metabolic demand has increased considerably in cows (1). Dry matter intake and the imbalance between energy demand and supply results in NEB in dairy cows (2, 3). To relieve NEB, dairy cows initiate lipid mobilization. However, this also causes negative effects, such as increasing the metabolic burden of the liver and risk of increased liver fatty acid levels, which leads to oxidative stress and immune dysfunction, thus influencing the health and lactation performance of cows (4, 5).

Leucine (Leu), valine (Val), and isoleucine (Ile) are the branched-chain amino acids (BCAA), and account for 50% of the essential amino acids (EAA) in milk proteins (6). Leu and lysine are the two most restrictive EAA in early lactation according to the concentration disparities of EAA in mammary arterial and venous blood (7). BCAA are important signaling regulatory molecules and nutrients that regulate protein synthesis via the activation of mTOR (8, 9), serve as precursors for the generation of non-essential amino acids, and regulate organismal energy metabolism processes by enhancing gluconeogenesis and mitochondrial oxidation in adipocytes. Many nutrients are degraded by rumen microorganisms. Rumen-protected BCAAs (RPBCAA) are used to reduce nutrient degradation and increase nutrient digestion and absorption in the small intestine. Supplementation with RPBCAA increases the contents of insulin (Ins) and glucose and reduces hyperketonemia and hepatic lipidosis (10, 11). Ins and glucose levels are also energy balance markers. In addition, BCAA catabolic byproducts can enter the tricarboxylic acid cycle to help meet the increased energy and nutrient requirements for milk production during early lactation (12–14). BCAAs are oxidized to produce ATP more efficiently than other amino acids and play important roles in cellular metabolism and stress responses. A strong association between BCAA concentrations and oxidative stress indicators has been observed (15). Wu et al. (16) showed that Leu and Ile protect MAC-T cells from H2O2-induced oxidative stress by regulating propionate metabolism.

Metabolomics has provided new perspectives for animal nutrition research through revealing the effects of external changes on metabolic pathways and biomarkers in ruminants (17–19). Several studies have used metabolomics to explore metabolite changes and biomarkers in milk and serum at different stages or under different nutritional conditions (20, 21). Their results have provided theoretical guidance for in-depth studies of the physiological status and nutritional utilization of cows.

Few studies examined the effects of RPBCAA supplementation on metabolic changes and mechanisms in the primiparous dairy cows. We hypothesized that feeding RPBCAA during early lactation would regulate energy and that lipid metabolism would mitigate oxidative stress and maintain the immune functions of cows, which also would be reflected in changes in certain blood and milk metabolites. Therefore, in combination with conventional physiological and biochemical indexes, the metabolic changes in early lactation dairy cows, as explored from the perspective of small molecules, were used to evaluate the effects of post-RPBCAA supplementation.

## 2 Materials and methods

### 2.1 Ethics statement

This study was performed in accordance with the Guidelines for the Care and Use of Experimental Animals of Jilin Agricultural University and was approved by the Jilin Agricultural University Laboratory Animal Welfare and Animal Experimental Ethical Inspection Committee (Jlau-acuc2021-005).

### 2.2 Experimental design and treatments

The study was performed at Yijiahe Dairy Farm (Ningxia Province, China) from December 20, 2022, to January 20, 2023. According to the gestation period (263.25 ± 2.79 d) and body condition score (BCS = 4.04 ± 0. 17), 20 primiparous (first birth) and healthy cows were selected 14 d before their expected calving date. Experiments were performed using a single-factor design. The cows were divided into two groups: control (CON) (n = 10), which received a basal diet only from calving to 21 d in milk, and RPBCAA (n = 10), which additionally received 95. 17 g/d of RPBCAA (44.60 g/d RPLeu, 25.14 g/d RPIle, and 25.43 g/d RPVal) and a basal diet from calving to 21 d in milk. Based on the NRC (2001) (22) prediction of the small-intestinal digestible amino acid content of each diet, amino acids were added achieve the corresponding amino acid level of the 17.00% CP high-protein diet. The actual per-rumen amino acid additions were calculated based on the product inclusion rate, ruminal degradation rate, and small-intestinal release rate. The RPBCAA was fed twice a day in equal amounts (4:30 and 13:30). The total mixed ration (TMR) was designed to meet the nutritional needs of cows during early lactation, according to the NRC (2001) (22). Before feeding with TMR, RPBCAA were mixed with a part of the TMR and provided to each cow in a small cattle trough for consumption; the remaining TMR was offered thereafter. All cows had free access to feed. During the experiment, the feeding environment was consistent between the RPBCAA and CON groups. Cows were fed TMR (4:30, 13:30, and 20:30) offered to achieve a 5% refusal rate, had free access to water, and were milked prior to feeding thrice daily; the milk yield was recorded daily.

RPBCAA were purchased from Changchun Borui Technology Co., Ltd. (Changchun, China). RPBCAA had a RPLeu content of 43.00%, 83.70% rumen bypass protection, and 74.74% intestinal digestibility; the RPIle content was 48.80%, with 73.38% rumen bypass protection, and 73.35% intestinal digestibility. The RPVal content was 54.40%, with 79.15% rumen bypass protection and 74.96% intestinal digestibility. The carriers of rumen amino acids were cellulose and starch. The intestinal digestibility data were determined according to the methods of Gargallo et al. (2006) (23).

### 2.3 Sample collection

Throughout the experiment, weekly samples of TMR were collected from the farm and stored at −20 °C. The starch, crude protein, dry matter, crude fat, acid detergent fiber, and neutral detergent fiber contents of the diets were assessed. TMR samples were thawed and dried at 105°C for 24 h to determine absolute dry matter (DM). Then, the samples were heated at 55°C for 48 h in a forced-air oven and stored in a dryer at 22 °C. All samples were assessed for DM (method 934.01; AOAC, 2005) (Shanghai Yiheng Scientific Instrument Co., Ltd, Shanghai, China), starch (method 996. 11; AOAC, 2005) (Shanghai Yidian Physical Optical Instrument Co., Ltd, Shanghai, China), crude proteins (method 954.01; AOAC, 2005), crude fat (method 920.39; AOAC, 2005), neutral detergent fiber (Van Soest et al., 1991), and acid detergent fiber (Van Soest et al., 1991) (Ankom Technology Corp., Fairport, NY, USA) with drying for 3 h at 105°C (24). The composition and nutritional parameters of the experimental diets are presented in **Table 1**. Net energy was calculated according to the NRC (2001) (22). BCS (5-point scale: 1 = thin, 5 = fat) was determined independently by two veterinarians on the 7 d before calving and 7, 21, and 45 d after calving date (25).

**Table 1:** Ingredients and nutrient composition of the basal diet in the early lactation dairy cowst (% of DM).

Ingredient	Content
Alfalfa hay	9.55
Corn	18.88
Flaked corn	7.11
Corn silage	32.56
Cottonseed	2.44
Cottonseed meal	5.86
Soybean meal	7.27
Extruded soybean	3.04
Megalac	1.57
DDGS^2^	3.91
Molasses	3.58
Corn gluten meal	1.52
5% Latation premix^3^	2.71
Nutrient levels^4^ (%, DM)	
CP	15.1
EE	3.76
NDF	30.16
ADF	17.63
Strach	29.68
Ca	0.80
P	0.38
NE_L_^5^ (mCal/kg)	1.78
RDP^6^ (%CP)	63.01
RUP (%CP)	36.99

^1^ DM, dry matter.

^2^ DDGS, distiller’s dried grain with solubles.

^3^ Provided per kilogram of total mixed ration (on DM basis): VA 516 KIU, VD 106 KIU, VE 6814 IU, nicotinamide 6815 IU, Cu 592 mg, Zn 2353 mg, Mn 1650 mg, Co 16.1 mg, I 25.7 mg, Se 34.0 mg.

^4^ Measured values.

^5^ Estimated based on NRC (2001). NE_L_ = Net energy required for lactation.

^6^ RUP and RDP calculated with CPM-Dairy.

Blood was sampled from the coccygeal vein using 20-gauge × 2.54 cm needles before morning feeding at 4:00 on d 21 relative to the actual calving date. Samples were stored separately in 5 mL heparin sodium anticoagulant-containing evacuated tubes and clot activator tubes. Blood collection tubes were centrifuged at 4000 × *g* for 15 min in 4°C conditions (AvantiJ-26XPI, USA). Serum samples were stored at − 80°C before analysis. The cows were milked three times per day at 5:00, 12:00, and 21:00, and milk production was measured daily using milking equipment (side-by-side parallel stall construction, Afimilk Ltd. Kibbutz Afikim, Israel). Samples of milk were collected thrice consecutively on days 7, 14, and 21 postpartum and were mixed in a ratio of 4:3:3 for each milking; 100 mL milk was collected from each cow, 0.5 mL of 20% potassium dichromate was added as a preservative, and the samples were stored in 2 × 50 mL centrifuge tubes at −40 °C. Samples were analyzed for milk fat, protein, lactose, and total solids using an infrared milk analyzer (Ekomilk Bond, Bulgaria), according to the manufacturer’s instructions.

### 2.4 Blood sample analysis

Serum samples were measured using ELISA kits obtained from the Shanghai Enzyme-linked Biotechnology Co., Ltd, and operating procedures were strictly followed. The concentrations of reactive oxygen species (ROS), total antioxidant capacity (T-AOC), superoxide dismutase (SOD), glutathione peroxidase (GSH-Px), malondialdehyde (MDA), catalase (CAT), non-esterified fatty acids (NEFA), β-hydroxybutyric acid (BHBA), glucose, insulin (Ins), glucagon (GC), insulin-like growth factor 1 (IGF-1), leptin, growth hormone (GH), very low-density lipoprotein (VLDL), acetyl coenzyme A carboxylase (ACC), fatty acid synthase (FAS), hormone sensitive lipase (HSL), triglyceride (TG), triglyceride lipase (ATGL), and immunoglobulin G (IgG), immunoglobulin A (IgA), immunoglobulin M (IgM), complement 3 (C3), complement 4 (C4) were determined using ELISA kits obtained from the Shanghai Enzyme-linked Biotechnology Co., Ltd. Absorbance was detected at 450 nm using a microplate reader (Scientific Instrument Co. Ltd., Shanghai, China). The inter- and intra-plate coefficients of variation were <5%.

### 2.5 Serum and milk metabolites

Untargeted metabolomics analyses of milk and plasma samples were performed using a Metabolomics Platform at Nanjing Jisi Huiyuan Biotechnology Co.(Nanjing, China). Gas chromatography–mass spectrometry (GC-MS) was used to measure milk and blood metabolites. Serum and milk samples collected at 21 d postpartum were analyzed using non-targeted metabolomics. Each 100 μL thawed sample was added to a 1.5 mL Eppendorf tube, and 400 µL of extract solution ethanol:acetonitrile = 1:1 with the isotopically labeled internal standard mixture) was added. All samples were sonicated in an ice water bath for 10 min. Then, they were placed in an ice refrigerator at −40 °C for 1 h, and the microcentrifuge tubes were centrifuged at 12,000 × *g* at 4°C for 15 min. The supernatant was transferred to a new glass microcentrifuge tube. Quality control samples were prepared by mixing equal aliquots of supernatants from all samples. Liquid chromatography–mass spectrometry (LC-MS) was performed using the Vanquish system (Thermo Fisher Scientific, Waltham, MA, USA) with the Water ACQUITY UPLC BEH Amide column (2. 1 mm × 50 mm, 1.7 µm) coupled to an Orbitrap Exploris 120 mass spectrometer (Orbitrap MS, Thermo Fisher Scientific). Mobile phase A consisted of 25 mM ammonium acetate and 25 mM ammonium hydroxide in water, whereas mobile phase B consisted of acetonitrile. The sample injection volume was 2 µL, and the auto-sampler temperature was 4 °C. A QE 120 mass spectrometer (Orbitrap MS, Thermo Fisher Scientific) was employed to acquire the tandem mass spectrometry spectra in the information-dependent acquisition mode under the control of the acquisition software (Xcalibur, Thermo Fisher Scientific). The specific parameters were as follows: sheath gas flow rate: 50 Arb, Aux gas flow rate: 15 Arb, capillary temperature: 320 °C, full MS resolution: 60000, MS/MS resolution: 15000, collision energy: SNCE 20/30/40, and spray voltage: 3.8 (positive) or −3.4 kV (negative).

### 2.6 Metabolite data acquisition

Basic data were converted into the mzXML form using the ProteoWizard software (https://proteowizard.sourceforge.io/), and recognition, extraction, alignment, and integration of the peaks were performed using the R package (XCMS as the core). The MS2 database was used to perform metabolite annotation. The cut-off for the annotation was set at 0.3. Data without a definite substance name, no spectral ratio, or substances with missing quantities greater than 50% in the comparison group were filtered and removed. For substances with less than 50% missing quantities, the K-nearest neighbor algorithm was used to simulate missing values. The total ion current or internal standard of each sample was used to normalize the data.

### 2.7 Statistical analyses

All experimental data, including BCS, lactation performance, and blood data, were analyzed using the SPSS software (version 26.0; SPSS Inc., Chicago, IL, USA). The CON and RPBCAA groups were analyzed using an independent sample t-test; data are presented as mean ± SEM. The significance level was set at *P* < 0.05.

A cluster tree analysis was performed on both sets of samples using the dendextend package in the R program (V3.6.2). The data were subjected to principal component and orthogonal partial least squares-discriminant analysis (OPLS-DA) using the SIMCA (V14. 1) software. Variable importance in projection scores were obtained from the OPLS-DA model. Supervised OPLS-DA was performed to acquire a high standard of group separation and to gain an improved understanding of the variables responsible for categorization. The significance of differences was calculated using Student’s *t*-test. Differential metabolites were screened using multivariate and univariate statistical analyses, provided that *P* was less than 0.05 and the variable importance in projection was greater than 1. A pathway function analysis of differential metabolites was conducted using the KEGG PATHWAY database (https://www.kegg.jp/kegg/pathway.html).

## 3 Results

### 3.1 Lactation performance and body condition

**Figure 1:**
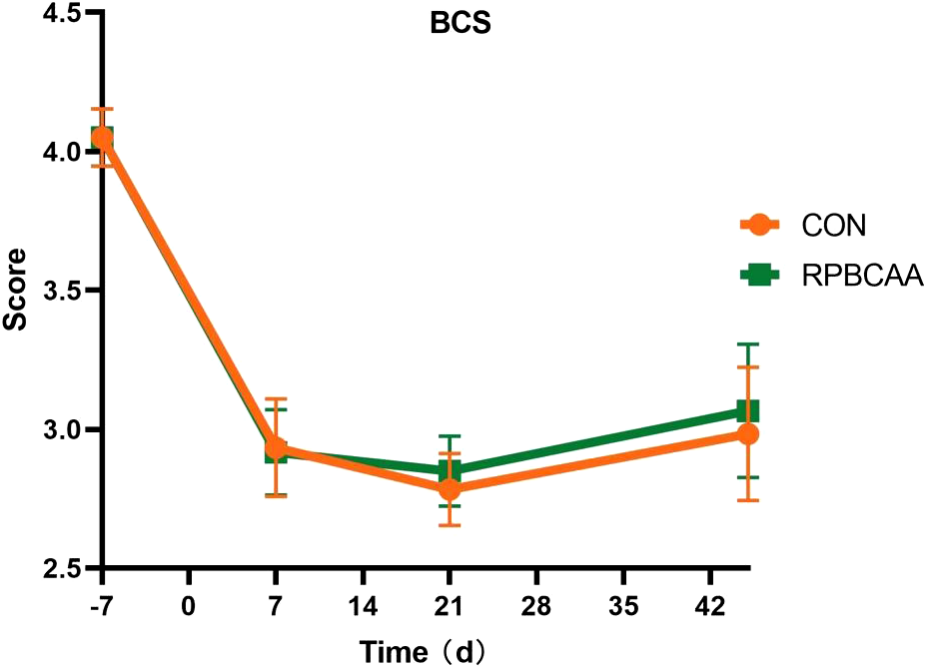
Body condition scores on the 7 d before calving and 7, 21, and 45 d after calving (CON, control; RPBCAA, rumen-protected branched chain amino acids).

The BCS is shown in **Figure 1**. Milk yields and compositions are summarized in **Table 2**. Feeding with RPBCAA did not affect the BCS (*P* > 0.05) or postpartum milk yield (*P* > 0.05). Throughout the experiment, RPBCAA supplementation had no effect on milk fat (*P* > 0.05), lactose (*P* > 0.05), proteins (*P* > 0.05), or total solids (*P* > 0.05).

**Table 2:** Effect of RPBCAA infusions on milk production parameters, milk yield, and body condition score during the postpartum period in Holstein dairy cows.

Item	Treatments^1^		SEM^2^	P-value
	CON	RPBCAA		
Milk yield, kg/d	26.88	27.62	0.22	0.10
Milk composition,%				
Fat	3.61	3.55	0.11	0.77
Protein	3.32	3.37	0.04	0.53
Lactose	4.84	4.90	0.06	0.62
Total solids	9.03	9.15	0.10	0.52

^1^Treatments: CON, control; RPBCAA, rumen-protected branched-chain amino acids.

^2^SEM, Standard error of the mean.

### 3.2 Serum parameters associated with energy metabolism

The serum biochemical parameters associated with energy intake are shown in **Table 3**. Postpartum serum NEFA (*P* > 0.05), BHBA (*P* > 0.05), leptin (*P* > 0.05), and glucose (*P* > 0.05) concentrations were unaffected by RPBCAA supplementation. However, the concentrations of Ins (*P* = 0.01), GC (*P* < 0.01), IGF- 1 (*P* = 0.02), GH (*P* = 0.02), and VLDL (*P* = 0.02) were greater in the serum of the RPBCAA group postpartum compared to those in the CON group. Two groups exhibited no significant differences in NEFA and BHBA contents.

**Table 3:** Effect of RPBCAA on energy and lipid metabolites during the postpartum period in Holstein dairy cows.

Item	Treatments^1^		SEM^2^	P-value
	CON	RPBCAA		
NEFA, μmol/ml	1015.04	1006.12	18.90	0.82
BHBA, μmol/ml	783.51	802.37	19.41	0.63
INS, mU/L	30.34	33.08	0.76	0.01
IGF-1, ng/ml	262.71	288.60	5.31	0.02
GH, ng/ml	21.33	23.46	0.47	0.02
GC, pg/ml	359.07	420.45	5.84	<0.01
Glu, ng/ml	56.54	56.03	0.74	0.73
Leptin, ng/ml	23.66	22.51	0.38	0.14
VLDL, mmol/ml	15.12	16.42	0.27	0.02
TG, mmol/ml	12.52	13.15	0.23	0.11
HSL, ng/ml	10.17	11.02	0.18	0.02
ATGL, ng/ml	208.14	219.01	3.49	0.13
FAS, nmol/mlm	16.72	19.69	0.29	<0.01
ACC, ng/ml	23.86	25.39	0.37	0.04

^1^Treatments: CON, control; RPBCAA, rumen-protected branched-chain amino acids.

^2^SEM, Standard error of the mean.

### 3.3 Serum parameters associated with lipid synthesis and decomposition

The effects of RPBCAA supplementation on fat synthesis and decomposition are shown in **Table 3**. During the postpartum period, no treatment effects were observed on the concentrations of serum TG (*P* > 0.05) and ATGL (*P* > 0.05) in the CON group. Additionally, compared with the CON group, the RPBCAA group showed increased concentrations of serum FAS (*P* < 0.01), HSL (*P* = 0.02), and ACC (*P* = 0.04) after calving.

### 3.4 Serum parameters associated with oxidative stress

The malondialdehyde content was less (*P* < 0.01) and glutathione peroxidase (GSH-Px) content was greater (*P* = 0.03) in the serum of postpartum cows in the RPBCAA group compared to those in the CON group (**Table 4**). The concentrations of T-AOC (*P* > 0.05), SOD (*P* > 0.05), CAT (*P* > 0.05), and ROS (*P* > 0.05) were not affected.

**Table 4:** Effect of RPBCAA on oxidative stress and immunity indicators during the postpartum period in Holstein dairy cows.

Item	Treatments^1^		SEM	P-value
	CON	RPBCAA		
ROS, U/ml	893.23	872.78	15.95	0.52
MDA, nmol/ml	12.03	11.02	0.15	<0.01
GSH-Px, ng/ml	761.43	825.10	14.06	0.03
SOD, ng/ml	11.05	11.00	0.20	0.92
T-AOC, pg/ml	383.55	391.37	4.81	0.42
CAT, ng/ml	213.61	207.12	2.30	0.17
IgA, μg/ml	2983.97	3132.84	57.08	0.16
IgG, μg/ml	8.61	9.65	0.20	<0.01
IgM, μg/ml	2435.31	2323.25	38.11	0.15
C_3_, μg/ml	76.23	77.35	3.47	0.88
C_4_, μg/ml	192.96	222.98	13.72	0.31

^1^Treatments: CON, control; RPBCAA, rumen-protected branched-chain amino acids.

^2^SEM, Standard error of the mean.

### 3.5 Serum parameters associated with immune function

Data corresponding to immune functions are shown in **Table 4**. The concentrations of IgA (*P* > 0.05), IgM (*P* > 0.05), C3 (*P* > 0.05), and C4 (*P* > 0.05) did not differ between the RPBCAA and CON groups. Furthermore, serum IgG concentrations were reduced by RPBCAA (*P* < 0.01).

### 3.6 Effect of dietary RPBCAA supplementation on serum metabolite profiles

**Figure 2:**
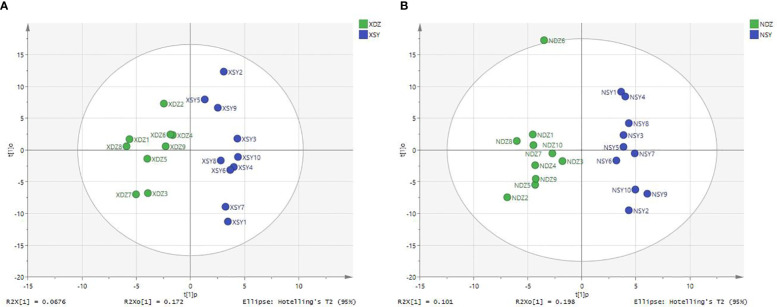
Plots of orthogonal partial least squares-discriminant analysis for serum **(A)** and milk **(B)** samples obtained from each dairy cow in the control (XDZ/NDZ) and treatment (XSY/NSY; received additional 95.17 g/d rumen-protected branched-chain amino acids) groups.

**Figure 3:**
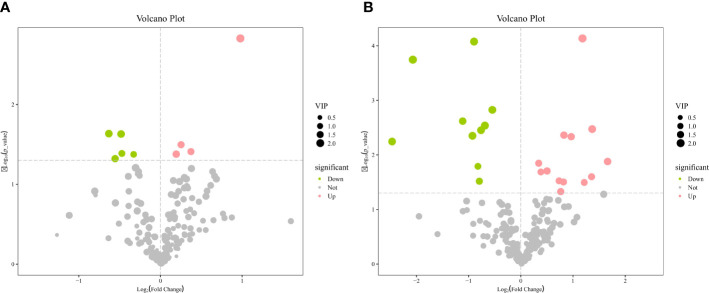
Volcano plots of metabolites in serum **(A)** and milk **(B)**.

**Figure 4:**
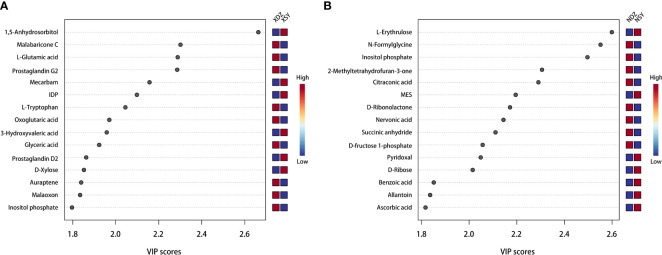
Top 15 serum **(A)** and milk **(B)** metabolites contributing to the variation in the first component of the partial least squares-discriminant analysis, shown by variable importance in projection scores in the control (XDZ/NDZ) and treatment (XSY/NSY; received additional 95.17 g/d rumen-protected branched-chain amino acids) groups.

The OPLS-DA method was effective in removing effects that were not relevant to the study. According to the OPLS-DA score plots, there was a clear separation between the CON and RPBCAA groups, suggesting that RPBCAA treatment led to different metabolic profiles in serum (**Figure 2A**) and milk (**Figure 2B**). A total of 414 metabolites were tested in the serum samples. Twenty-three blood differential metabolites were identified based on the OPLS-DA model (**Figure 3A**). Leu-Leu-OH, leucyl-tryptophan, LysoPC [P- 18:1 (9Z)], citrulline, SM [d17:1/24:1 (15Z)], 1-(Hydroxymethyl)-5,5-dimethyl-2,4-imidazolidinedione, N-Acetyl-L-arginine, phosphatidylcholine (PC) [P- 18:1 (9Z)/14:1 (9Z)], urocanic acid, and PC [P- 18:1 (11Z)/20:0] were the 10 most variable metabolites at 21 d (*P* < 0.05) (**Figure 4A**).

**Figure 5:**
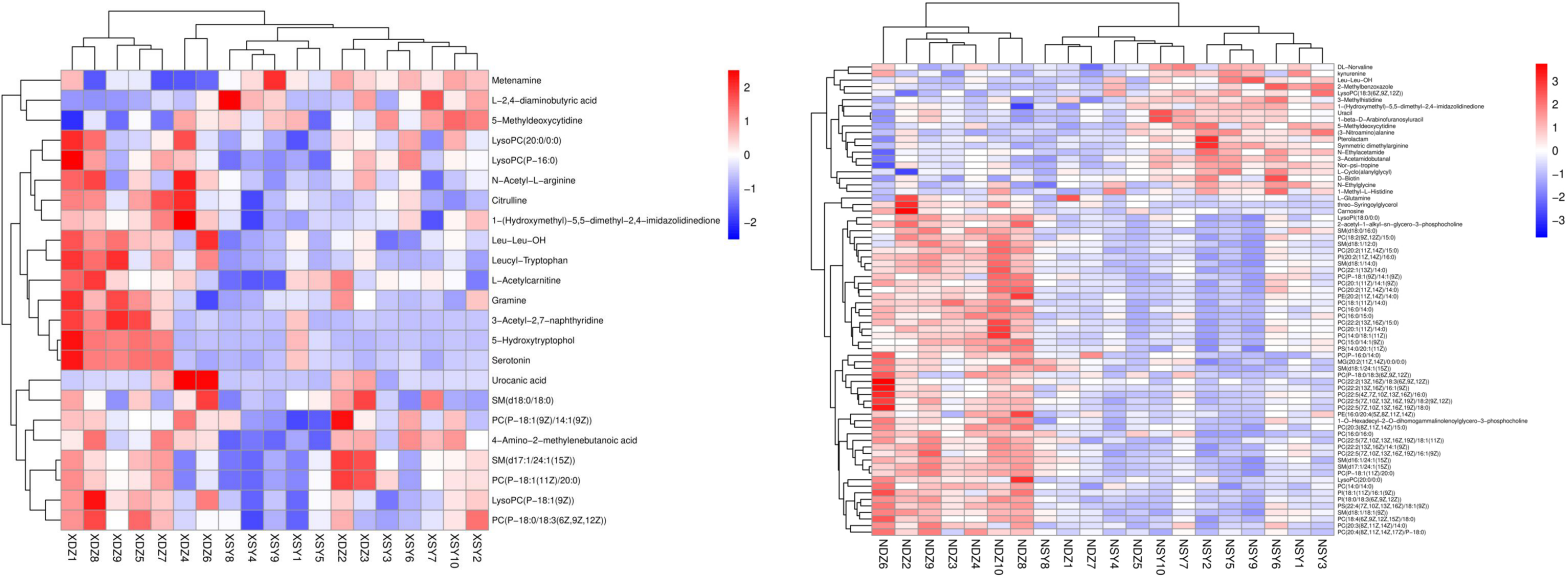
Heatmap of the hierarchical clustering analysis for metabolites that were present at different concentrations in the control (XDZ/NDZ) and treatment (XSY/NSY; received additional 95.17 g/d rumen-protected branched-chain amino acids) groups (P < 0.05, variable importance in projection >1.0).

**Figure 6:**
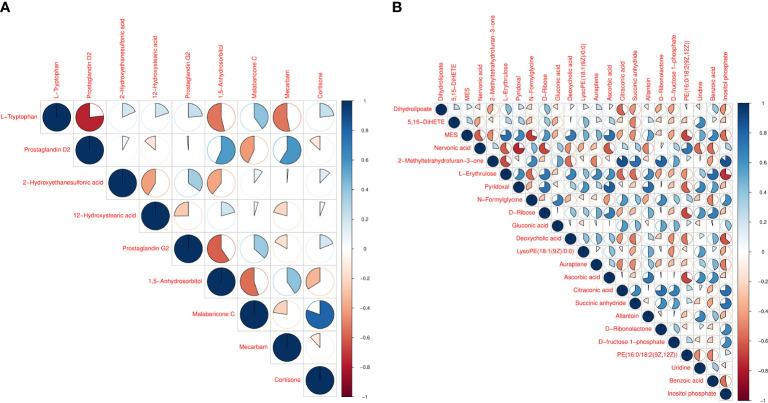
Vertical and diagonal representation of serum **(A)** and milk **(B)** differential metabolites. The colors represent the level of correlation between the different metabolites - the darker the color the higher the correlation.

**Figure 7:**
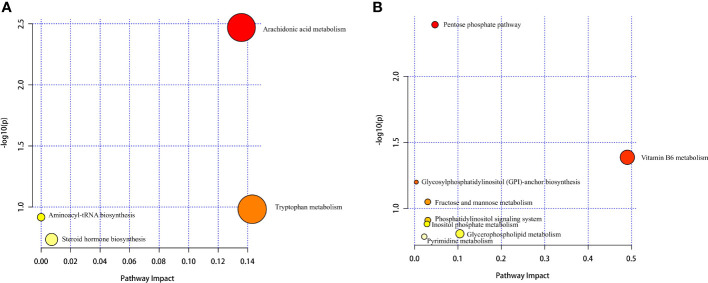
Serum **(A)** and milk **(B)** metabolomics view maps of the significant differential metabolites in the control and treatment groups. The x-axis represents the pathway impact, and the y-axis is the value of −log (P). The size represents pathway enrichment, and the color indicates pathway impact values, with darker color indicating higher values.

Hierarchical cluster analysis of the differential metabolites in the serum showed that metabolites with similar changes converged in the same cluster (**Figure 5A**). **Figure 6A** shows the results of differential metabolite association analysis. Quantitative enrichment analysis identified five metabolic pathways: arginine biosynthesis and histidine, sphingolipid, glycerophospholipid, and tryptophan metabolism (**Figure 7A**).

### 3.7 Effect of dietary RPBCAA supplementation on milk metabolite profiles

A total of 430 metabolites were tested in the milk samples. Seventy-two differential metabolites were identified based on the OPLS-DA model (**Figure 3B**). LysoPC [18:3 (6Z,9Z, 12Z)], SM [d16:1/24:1 (15Z)], PC [P- 18:1 (11Z)/20:0], SM [d17:1/24:1 (15Z)], threo-Syringoylglycerol, 1-O-Hexadecyl-2-O-dihomogammalinolenoylglycero-3-phosphocholine, PC (16:0/14:0), PC [20:3 (8Z, 11Z, 14Z)/15:0], PC [20:2 (11Z, 14Z)/15:0], and PC (16:0/16:0) were the 10 most variable metabolites at 21 d (**Figure 4B**). The abundance of LysoPC [18:3 (6Z,9Z, 12Z)] in the RPBCAA group was lower than that in the CON group, whereas the relative abundances of the remaining nine metabolites increased.

The hierarchical cluster analysis of the differential metabolites in milk showed that metabolites with similar changes converged in the same cluster (**Figure 5B**). **Figure 6B** shows the results of differential metabolite association analysis. Quantitative enrichment analysis identified five metabolic pathways: glycerophospholipid, histidine, beta-alanine, pyrimidine, and linoleic acid metabolism (**Figure 7B**).

## 4 Discussion

Dairy cows undergo considerable physiological and immunological changes as they transition from pregnancy to lactation, with the incidence rate of metabolic disorders being the highest during early lactation (26). Following parturition, cows do not eat a sufficient amount, making it difficult or impossible to meet their increased nutritional requirements. This phenomenon results in the mobilization of their fat reserves and muscle tissue to compensate for malnutrition.

### 4.1 Lactation performance and body condition

Grummer et al. (27) reported that decreased DMI and accompanying high energy demand led to NEB, which can reduce cow performance and trigger energy metabolic diseases in dairy cows. Therefore, early lactation is a critical phase requiring intensive dietary management.

In the current study, the milk yield and composition were not significantly different following RPBCAA supplementation during early lactation. This result is consistent with that of Yepes et al. (10). However, Rulquin and Pisulewski (28) demonstrated marked milk protein yield responses when 40 g/d of Leu or more was infused via the duodenum of the cows. The distribution of EAA changes during anabolism and catabolism in organisms (29) and the oxidation of amino acid in the liver, muscles, and other tissues may be limiting factors in mammary protein synthesis (30). Milk production and composition differences are determined via differentiation, including the level of BCAA additive, stage of addition time, amino acid ratio, and nutritional level in the diets (7, 31). Moreover, RPBCAA supplementation did not affect milk composition in the present study, and the differences in the results from these studies may be related to the mammary tissue ingestion of BCAA, which are used more for breast tissue development than for synthesizing milk components (32). Supplementation with RPBCAA had no marked effect on body condition ratings.

### 4.2 Serum variables related to energy metabolism

Excessive serum concentrations of NEFA and BHBA in dairy cows are commonly used as indicators of poor metabolic status and NEB during early lactation (33). A serum NEFA concentration of 1.00 mmol/L can cause ketosis (34). In the current study, the serum NEFA concentration of the cows was higher than 1.00 mmol/L, indicating that the cows were in a high-NEB status at this time. High serum levels of NEFA interfere with the Ins signaling pathway and reduce Ins sensitivity (35).

Ins and GC are important energy metabolism-regulating hormones in animals. These hormones help regulate the metabolism of three major substances and play a major role in maintaining the stability of energy metabolism. In the current study, Ins and GC were elevated in the RPBCAA group. This may have resulted from BCAA promoting Ins secretion via Vagus nerve-dependent mechanisms (36, 37). As ketogenic and gluconeogenic amino acids, BCAA produce glucose during catabolism. The concentrations of blood glucose were increased, which stimulated the release of insulin. IGF- 1 and Ins are homologous and improve the cellular uptake and utilization of glucose (38, 39). They also promote the uptake and utilization of glucose in tissues, which accelerates the increase in GC. However, the blood glucose concentration in the RPBCAA and CON groups was unchanged in the present study. This result indicated that RPBCAA maintain energy balance and promote energy metabolism in cows.

### 4.3 Serum variables related to lipid synthesis and decomposition

The levels of fatty acid synthases and catabolic enzymes were considerably great in RPBCAA group in the present study. Furthermore, fat synthesis and catabolism were in a highly dynamic state of equilibrium. VLDL transports endogenous triglycerides synthesized in the liver to the extrahepatic compartment, and a slow output of lipids in the form of VLDL results in a fatty liver (40, 41). However, in the present study, the supplementation with RPBCAA increased the activity of ACC, FAS, and HSL. This may have resulted from the promotion of Ins secretion. However, this lipolytic effect may be attributed to elevated GC levels. In dairy cows, the balance between adipose lipolysis and lipogenesis is pivotal for maintaining lactation and is regulated by numerous factors, including the lactation stage, energy intake, and production level (42).

### 4.4 Serum variables related to oxidative stress, immune function

Accelerated metabolism and the mobilization of body tissues in periparturient cows as well as postpartum uterine recovery and initiation of lactation are often accompanied by the onset of oxidative stress. During this period, ROS accumulate in the organisms (43), and excessive ROS levels lead to cellular oxidative stress (44). The role of BCAA in alleviating oxidative stress in ruminants has been widely recognized (15, 16, 45). BCAAs promote the expression of antioxidant enzymes, increase the cellular energy supply, enhance peroxisomal and mitochondrial functions, and improve antioxidant capacity (46). The cell membranes of immune cells contain a high concentration of unsaturated fatty acids that are highly sensitive to peroxidation. Therefore, the immune function of immune cells stimulated by excess free radicals decreases, which in turn affects the functions of the immune system (47). Therefore, RPBCAA supplementation may alleviate oxidative stress, reduce lipid peroxidation, and maintain IgG levels in lactating dairy cows.

### 4.5 Milk and serum metabolomics profiling

To further explore the metabolic mechanism through which RPBCAA supplementation alleviates NEB in early lactating dairy cows, LC-MS was used to evaluate serum and milk metabolites. The pathway analysis of serum and milk consistently indicated a major impact of amino acid and lipid metabolism on these pathways. Amino acid metabolism is crucial for protein synthesis and other biosynthetic reactions. In the present study, the blood showed significant differences in metabolites enriched in histidine metabolism and the arginine biosynthesis pathway. Additionally, glycerophospholipid and linoleic acid metabolism pathways were significantly affected in milk.

Multiple amino acid biomarkers were detected in the serum of cows. 5-Hydroxytryptophan is also known as serotonin. Serotonin mediates liver regeneration and regulates glucose and Ins homeostasis (48). Laporta et al. (49) showed that increasing 5-hydroxytryptamine levels during the transition from gestation to lactation increased the mRNA expression of enzymes involved in liver energy metabolism, mRNA abundance, and distribution of glucose transporters in the mammary gland and regulated the energy metabolism of mammary tissue. Their result is physiologically consistent with our observation of elevated insulin levels and increased energy metabolism. The glutamine abundance was higher in the RPBCAA group than in the CON group. Glutamine is a precursor for synthesizing the antioxidant glutathione, which is an important molecule in the protection of cells against oxidative stress and can strengthen the immune system. Xu et al. (50) found that BCAA supplementation may improve the abundance of serum glutathione during early lactation. Our study suggests that the addition of RPBCAA may have improved the antioxidant status, reduced oxidative damage, and maintained the immune function of the organism. PC is an essential component of VLDL synthesis (51). VLDL transports unoxidized NEFA out of the liver (25) and can prevent liver fat deposition as well as reduce the incidence of fatty liver in cows. This is consistent with the elevated plasma VLDL levels in our study. Breast epithelial cells can recognize and uptake VLDL from the bloodstream (52). However, due to increased abundance of milk PC, we hypothesized that RPBCAA supplementation catalyzed PC metabolism predominantly in the milk gland.

The untargeted metabolomic analysis provided a comprehensive map of metabolites and related pathways that revealed a possible relationship between RPBCAA supplementation and amino acid synthesis, metabolism, and lipid metabolism. The differences in metabolic pathways and products will form the basis of future research on dairy cattle metabolism. Currently, few studies have explored the role of BCAA in periparturient dairy cows. The current study demonstrated that RPBCAA can alleviate NEB in periparturient cows. However, tissue samples could not be collected to perform an in-depth study of the mechanism. The results for non-targeted metabolites suggest that BCAA are highly relevant to lipid metabolism. Supplementary validation tests could be conducted to further investigate these results.

## 5 Conclusion

In summary, adding BCAAs to the diet can improve metabolic levels of the dairy cow body by influencing endocrine functions and improving the cows’ energy metabolism, thus regulating amino acid, and lipid metabolism. Concurrently, it can mitigate oxidative stress and maintain the immune function of cows. Future studies should focus on the use of RPBCAA as supplements for dairy cows to fully detail the mechanism of BCAA effects on energy and lipid metabolism.

## Data availability statement

The data analyzed in this study is subject to the following licenses/restrictions: The data are not publicly available due to restrictions their containing information that could compromise the privacy of research participants. Requests to access these datasets should be directed to Zhikun Zhao, zhaozhikun2021@163.com.

## Ethics statement

The animal study was approved by all experimental procedures were performed in accordance with the Guidelines for the Care and Use of Experimental Animals of the Jilin Agricultural University (Jlau-acuc2021-005). The study was conducted in accordance with the local legislation and institutional requirements. Written informed consent was obtained from the owners for the participation of their animals in this study.

## Author contributions

ZZ: Data curation, Formal analysis, Methodology, Validation, Writing – original draft, Writing – review & editing. JD: Validation, Writing – review & editing. DW: Project administration, Writing – review & editing. CZ: Investigation, Writing – review & editing. XT: Software, Writing – review & editing. YM: Conceptualization, Writing – review & editing. YZ: Investigation, Writing – original draft. YZ: Investigation, Writing – review & editing. GQ: Supervision, Writing – review & editing. TW: Visualization, Writing – review & editing. ZS: Visualization, Writing – review & editing. XZ: Writing – review & editing, Methodology, Project administration. YZ: Writing – review & editing, Resources.
